# Targeting GRP78-dependent AR-V7 protein degradation overcomes castration-resistance in prostate cancer therapy

**DOI:** 10.7150/thno.41849

**Published:** 2020-02-10

**Authors:** Yuning Liao, Yuan Liu, Xiaohong Xia, Zhenlong Shao, Chuyi Huang, Jinchan He, Lili Jiang, Daolin Tang, Jinbao Liu, Hongbiao Huang

**Affiliations:** 1 Affiliated Cancer Hospital & institute of Guangzhou Medical University, Protein Modification and Degradation Key Lab of Guangzhou and Guangdong, State Key Laboratory of Respiratory Disease, School of Basic Medical Sciences, Guangzhou Medical University, Guangzhou, Guangdong 510095, China; 2 Department of Surgery, UT Southwestern Medical Center, Dallas, Texas 75390, USA

**Keywords:** AR-V7, CRPC, rutaecarpine, GRP78, SIAH2

## Abstract

**Rationale**: Androgen receptor splice variant 7 (AR-V7) is a leading cause of the development of castration-resistant prostate cancer (CRPC). However, the regulation and function of AR-V7 at levels of post-translational modifications in prostate cancer therapy remain poorly understood. Here, we conducted a library screen of natural products to identify potential small molecules responsible for AR-V7 protein degradation in human prostate cancer cell lines.

**Methods**: A natural product library was used to screen the inhibitor of AR-V7. Co-IP and biomass spectrum assays were used to identify the AR-V7-interacting proteins, whereas western blot, confocal microscopy, RNA interfering, and gene transfection were used to validate these interactions. Cell viability, EDU staining, and colony formation assays were employed to detect cell growth and proliferation. Flowcytometry assays were used to detect the distribution of cell cycle. Mouse xenograft models were used to study the anti-CRPC effects *in vivo*.

**Results**: This screen identified rutaecarpine, one of the major components of the Chinese medicine *Evodia rutaecarpa*, as a novel chemical that selectively induces AR-V7 protein degradation via K48-linked ubiquitination. Mechanically, this effect relies on rutaecarpine inducing the formation of a GRP78-AR-V7 protein complex, which further recruits the E3 ligase SIAH2 to directly promote the ubiquitination of AR-V7. Consequently, the genetic and pharmacological activation of the GRP78-dependent AR-V7 protein degradation restores the sensitivity of castration-resistant prostate cancer to anti-androgen therapy in cell culture and animal models.

**Conclusions**: These findings not only provide a new approach for overcoming castration-resistance in prostate cancer therapy, but also increase our understanding about the interplay between molecular chaperones and ubiquitin ligase in shaping protein stability.

## Introduction

Prostate cancer (PC) is a common malignancy with high incidence and mortality among males worldwide 1. The endocrine therapies that aim to block androgen-androgen receptor (AR) signaling via depriving androgen or its binding to AR, therapies such as the use of abiraterone and enzalutamide (Enza), have been a great success in certain patients with advanced and metastatic PC. However, 20%-40% of patients with PC who do not respond to endocrine therapies, and many other patients who initially respond to the therapies, eventually develop secondary castration-resistant PC (CRPC) 2. Interestingly, AR signaling remains functional in CRPC 3, 4, but the underlying mechanisms of this remain poorly defined. Increasing evidence has demonstrated that AR splice variants (AR-Vs) not only act as biomarkers predicting prognosis, but also as drivers contributing to CRPC development 5-7.

Among multiple AR-Vs, AR-V7 is the most frequently expressed clinically, and is functionally relevant to CRPC development 6. Preclinical studies also demonstrate that AR-V7 promotes CRPC and mediates Enza resistance 8-10. Compared to AR, AR-V7 structurally retains the N-terminal (NTD), DNA-binding domain (DBD), but lacks the ligand-binding domain (LBD) that is replaced with variant-specific cryptic exon 3 (CE3) in the C-terminal 11. This AR isoform is androgen-independent and continuously activated 11. While the LBD is the target site of traditional endocrine therapies, the presence of AR-V7 may contribute to the AR reactivation in CRPC.

Although the significance of AR-V7 has been well-demonstrated, the regulatory network controlling AR-V7 protein stability remains to be fully understood. There is also no specific inhibitor of AR-V7 available in the clinic, which hinders investigations as well as treatments for CRPC. The current study aims to identify potential small molecules responsible for AR-V7 protein degradation and further uncover the underlying mechanisms of action of their biomedical effects on overcoming CRPC. Based on a library screen of natural products, we found that rutaecarpine (Rut), an alkaloid extracted from the Chinese medicine *Evodia rutaecarpa*, exhibited previously unknown activity in promoting AR-V7 protein degradation via the activation of the molecular chaperone glucose-regulated protein 78kD (GRP78, also known as BiP) and siah E3 ubiquitin protein ligase 2 (SIAH2). Importantly, targeting GRP78-dependent AR-V7 protein degradation via SIAH2 overcame CRPC *in vitro* and *in vivo*.

## Materials and Methods

### Materials

Chemicals: natural product library (#L1400), rutaecarpine (#S2349), parecoxib (#S4656), bortezomib (#S1013), MG132 (#S2619), enzalutamide (#S1250), and bicalutamide (#S1190) were obtained from Selleckchem (Houston, TX). Antibodies: anti-AR-V7 (#19672), anti-AR (#5153), anti-K48-ubiquitin (#12805), anti-IgG (#3900), anti-MDM2 (#86934), anti-CDK2 (#2546), anti-CDK4 (#12790), anti-CDK6 (#13331), anti-cyclinD1 (#2978), anti-p21 (#2947), anti-p27 (#3686), anti-p15 (#4822), anti-COX2 (#12282), anti-HA-tag (#2367), anti-FLAG-tag (#8146), anti-GAPDH (#5174), anti-GRP94 (#20292), anti-HSP90 (#4877), and anti-lamin B1(#13435) were obtained from Cell Signaling Technology (Beverly, MA); anti-GRP78 (#ab21685), anti-HSP7C (#ab51052), anti-SIAH2 (#ab230532), anti-STUB1 (#ab134064), anti-AR-V7 (#ab198394, for IHC), and anti-Ki67 (#ab15580) were obtained from Abcam (Cambridge, MA).

### Cell culture

The human prostate epithelial cell line WPMY-1; AR-positive PC cell lines LNCaP, C4-2, and 22Rv1; and AR-negative PC cell lines PC3 and DU145 were purchased from the American Type Culture Collection (Manassas, VA). The above cell lines were cultured in an incubator at 37 °C with 5% CO_2_, according to our previous reports 12, 13. WPMY-1 cells were cultured in DMEM with 10% FBS. 22Rv1, LNCaP, and C4-2 cells were cultured in RPMI-1640 with 10% FBS. PC3 and DU145 cells were cultured in DMEM/F12 with 10% FBS. Cell line identity was validated by short tandem repeat profiling, and routine mycoplasma testing was negative for contamination.

### RNA interfering

The RNA interfering assays using siRNA or shRNA were performed according to our previous reports 14, 15. Briefly, for siRNA transfection, exponentially growing 22Rv1 cells were seeded on the dish for 24 h. Then siRNAs targeting human GRP78 or control siRNA mixtures (10 μM), including lipofectamine RNAiMax (Invitrogen), and RPMI opti-MEM (Gibco), were prepared at a ratio of 10 μl: 10 μl: 480 μl and incubated for 15 min. The siRNA-lipofectamine mixtures were then added in each group for 48 h for further analysis. The GRP78 siRNAs (#sc-29338) were purchased from Santa Cruz Biotechnology (Santa Cruz, CA). The siRNA sequences are listed below (all are provided in 5' to 3' orientation): GRP 78 siRNA-1: sense: GUGGUGCCUACCAAGAAGUtt, antisense: ACUUCUUGGUAGGCACCACtt; GRP 78 siRNA-2: sense: GAAGAAUUGGCCAUCUUAAtt, antisense: UUAAGAUGGCCAAUUCUUCtt.

For lentivirus shRNA transfection, lentivirus (pLKD-CMV-Puro-U6-shRNA) containing 2 pairs of shRNAs targeting AR-V7 or non-specific sequences (control shRNAs) were obtained from GeneChem (Shanghai, China). Additionally, 22Rv1 cells were seeded in 6-well plates for 24 h, and replaced with fresh medium containing 5 μg/ml polybrene (Santa Cruz, CA). Lentiviruses were then added to the cells at a multiplicity of infection of 10. After incubation for 48 h, puromycin selection was performed to eliminate the unsuccessfully transfected cells at a concentration of 2 μg/ml. The sequences of AR-V7 shRNAs are listed below (all are provided in 5' to 3' orientation): AR-V7 shRNA-1: CcggCCGACTTTCCCTCTT ACATTTCAAGAGAATGTAAGAGGGAAAGTCGGTTTTTTg; AR-V7 shRNA-2: CcggGCCAGACTCAAATATTGTATTCAAGAGATACAATATTTGAGTCTGGCTTTTTTg.

### Plasmid transfection

The following plasmids were constructed and purchased from GeneChem: plasmids (CMV-MCS-3FLAG-SV40-neomycin) containing the full length of human GRP78 CDS (gene ID: 3309) and its truncated mutants or control plasmids; the plasmids (CMV-MCS-HA-SV40-neomycin) containing the full length of human AR-V7 CDS (gene ID: 367) and its mutant form that lacks ΔCE3 on its C-terminal (AR-V7ΔCE3) or control plasmids; and the plasmids (CMV-myc-MCS) containing human SIAH2 CDS (gene ID: 6478). Lipofectamine 3000 (Invitrogen) reagent, RPMI opti-MEM (Gibco), and the plasmids were prepared in the following ratio: P3000 (2 μl): plasmids (1 μg): lipofectamine 3000 (2 μl). After incubation for 15 min, the mixtures were added to the cells seeded on plates or dishes and remained there for 48 h for further analysis, and fresh medium was replaced appropriately. The final concentration of the plasmids was 0.75 μg/ml.

### Immunofluorescence assay

Immunofluorescence analysis was performed as previously described 16, 17. Briefly, 22Rv1 cells were transfected with HA-AR-V7 for 48 h. Cells were washed with cold PBS and then fixed with 4% paraformaldehyde for 15 min. Fixed cells were washed again and then permeabilized with 0.5% Triton-X for 5 min. After permeabilization, the cells were blocked with 5% bovine serum albumin (Sigma) for 30 min, and then antibodies as indicated in the figures were added and incubated overnight at 4°C. Anti-mouse IgG H&L (Alexa Fluor 488, Abcam) and anti-rabbit IgG H&L (Alexa Fluor 647, Abcam) secondary antibodies were used to generate green and red fluorescence. DAPI (0.1 μg/ml, Abcam) staining was used to visualize nuclei. A confocal microscope (Leica TCS SP8) was employed to image the cells.

### PCR assay

A PCR assay was used to determine the mRNA levels of AR-V7 in 22Rv1 cells exposed to Rut for 12 h, and this assay was performed as previously described 15. Briefly, total RNAs were extracted from 22Rv1 cells using RNAiso plus (TaKaRa Biotechnology, Dalian, China). The RNA purity and concentration were confirmed with a ratio of 260: 280 nm. The first-strand cDNA was synthesized with a PrimeScript RT Master Mix kit (TaKaRa, Dalian, China). Real-time quantitative PCR was used to measure the mRNA levels of AR-V7 by employing a SYBR Premix Ex TaqTM kit (TaKaRa, Dalian, China) according to the manufacturer's instructions. GAPDH was used as an internal control. The primers of AR-V7 and GAPDH were selected from a previous study 6; AR-V7 forward: 5'-CAGCCTATTGCGAGAGAGCTG-3', AR-V7 reverse: 5'-G AAAGGATCTTGGGCACTTGC-3'; GAPDH forward: 5'- TCCCATCACCATCTT CCA-3'; reverse: 5'-CATCACGCCACAGTTTCC-3'.

### Extraction of nuclear and cytoplasmic protein

This assay was performed according to the instructions of the Nuclear and Cytoplasmic Protein Extraction Kit (#KGP150, KeyGEN BioTECH, China). Briefly, 22Rv1 cells were digested and centrifuged for 5 min at 800 ×g at 4°C, and then washed with cold PBS twice. The premixed mixtures of protease inhibitor cocktails, buffer A and buffer B (working solution 1) were then added to the collected cells and placed on ice for 30 min. After centrifugation for 10 min at 3000 ×g at 4°C, the supernatant was collected and considered as cytoplasmic proteins. The sediment was washed with working solution 1 twice at 800 ×g at 4°C, After discarding the supernatant, the premixed mixtures of protease inhibitor cocktails and buffer C (working solution 2) were added to the pellet, and followed by ultrasonic treatment with a Vibra-Cell Ultrasonic Liquid Processor (Sonics, Newtown, CT) for 10 s and placed on ice for 30 min. After centrifugation at 14,000 ×g for 30 min at 4 °C, the supernatant was collected and considered as nuclear proteins. The extracted nuclear and cytoplasmic proteins were used in the following western blot and co-IP assays.

### Western blot assay

This assay was performed as described before 13. Briefly, equal amounts of extracted proteins were prepared with RIPA buffer, protease inhibitors (Cell Signaling Technology), and a BCA assay kit (Thermo Fisher). The proteins were mixed with 3× blue loading buffer (#7722, Cell Signaling Technology) and denatured in a boiling water bath (100 °C) for 5 min and then separated in 12% SDS-PAGE, and then transferred to polyvinylidene difluoride (PVDF) membranes. 5% milk was then used to block the blots for 1 h. Primary antibodies and horseradish peroxidase-conjugated secondary antibodies were each incubated for 1 h. The bound secondary antibodies were reacted to the ECL detection reagents and exposed to X-ray films (Kodak, Japan). PBS-T was used to wash the PVDF membranes 3 times during each incubation.

### Co-IP analysis

This assay was performed as described before 15. Briefly, cell lysates and extracted proteins were prepared with RIPA buffer and protease inhibitors. Dynabeads (Invitrogen) and antibody mixtures were prepared and incubated for 16 h. Extracted proteins were then mixed with the Dynabeads containing specific antibodies, and incubated on a rotator at 4 °C for 1 h. After incubation, the mixtures were washed for 3 times with PBS-T. The mixtures were then resuspended with SDS loading buffer, followed by a boiling water bath. After centrifugation, the Dynabeads were discarded and the supernatant was used for further analysis.

### Cell proliferation assays

Cell proliferation assays, including those for cell viability, colony formation, EDU staining and cell cycle, were performed as previously reported 18-20. MTS (Promega) assay was used to test the cell viability. Briefly, PC cells were seeded on 96-well plate for 24 h at a concentration of 2000 cells/well. Cells were treated as indicated in figure legends. MTS (20 μl) reagent was added in each well in the dark and cells were incubated for 3 h. The absorbance of optical density was measured with a microplate reader (Sunrise reader, Tecan, Mannedorf, Switzerland) at a wavelength of 490 nm from three independent experiments. The combination index was calculated using the Chou-Talalay equation as we previously reported 21. EdU (Ribobio, Guangzhou, China) assay was used to test the DNA reproduction rate. Briefly, PC cells were seeded on a chamber slide for 24 h, and then treated with Rut for 48 h. Cells were incubated with 50 μM EdU for 2 h. After incubation, cells were washed with PBS and fixed with 4% paraformaldehyde for 30 min. Cells were then incubated with glycine for 5 min, 0.5% Triton X-100 for 10 min, Apollo reaction cocktail for 30 min, and DAPI for 5min. Cells were washed with PBS during the above incubations. Images from three independent experiments were captured by an Olympus microscope.

PC cells were seeded in 60 mm dishes and treated with indicated doses of Rut for 48 h. Cells were then digested and washed with PBS. For clonogenic assays, PC cells were resuspended and re-seeded on a 6-well plate and cultured for 2 weeks. Cells were then fixed with 4% paraformaldehyde for 10 min, and stained with 1% crystal violet solution for 5 min. Colonies > 60 μm were counted from three independent experiments. For cell cycle assay, PC cells were washed with PBS twice and resuspended with 500 μl PBS and 2 ml 70% ethanol at 4 °C overnight. Cells were then washed with PBS and incubated with the reaction mixtures containing PI (50 μg/ml; Keygen, Nanjing, China), RNase A (100 μg/ml), and 0.2% Triton X-100 for 30 min at 4 °C. Cell cycle distributions of each group were ultimately analyzed with flow cytometry from three independent experiments.

### Animal models

All animal experiments were approved by institutional animal care and use committees. The 22Rv1 xenograft models were established as previously described 12. Briefly, nude Balb/c mice were bred at the animal center of Guangzhou Medical University. Exponentially growing 22Rv1 cells were prepared and inoculated subcutaneously on the flanks of 5- to 6-week-old male nude mice at a concentration of 2 × 10^6^ cells/100μl PBS/mice. After inoculation for 1 week, the mice were randomly divided into 9 groups (10 mice/group); 3 groups of them were treated with Rut at 20mg/kg/2d (i.p.) or 40mg/kg/2d (i.p.) or with vehicle for 15 days. Another 6 groups were used to determine the synergistic effects of Rut and Enza/Bica, and these mice treated with vehicle, Rut 20mg/kg/2d (i.p.), Enza 25mg/kg/2d (p.o.), Bica 25mg/kg/2d (p.o.), Rut + Enza, or Rut + Bica for 15 days. The nude mice sacrifice after 22 days of inoculation, the size and weight of tumors and body weight of mice were measured and calculated as reported previously 12.

### Statistical analysis

Data are presented as mean ± SD from three independent experiments with multiple determinations for each experiment where applicable. To determine statistical probabilities, unpaired Student's *t*-tests or one-way ANOVA is used where appropriate. Statistical analysis was performed with GraphPad Prism5.0 software and SPSS 16.0. A *p* value of < 0.05 was considered statistically significant.

## Results

### Rutaecarpine selectively promotes the K48-linked ubiquitination accumulation and degradation of AR-V7

**Figure 1:**
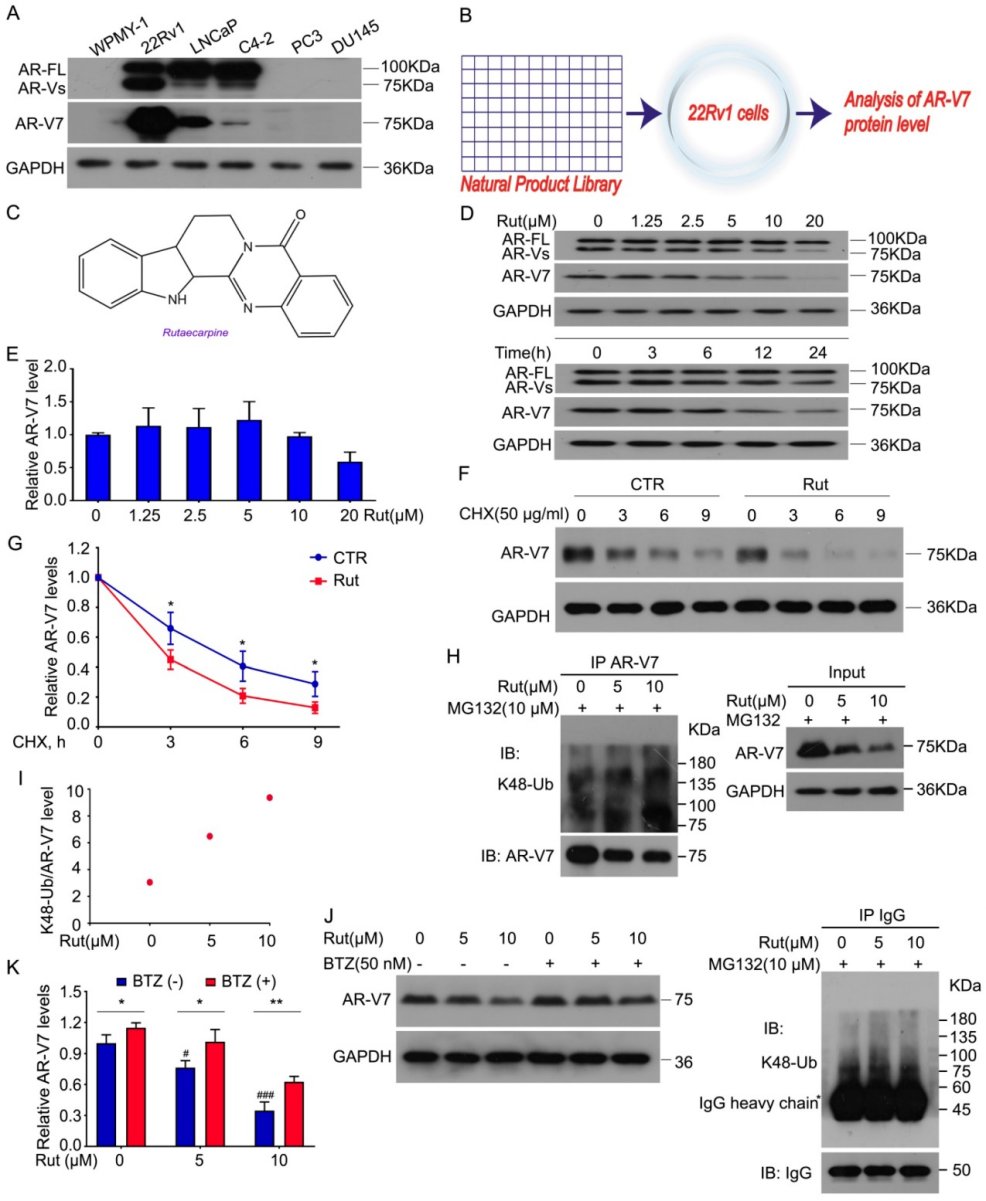
** Rutaecarpine stood out as a selective inhibitor of AR-V7 by promoting its degradation in proteasome**. **(A)** Immunoblot analysis of AR-FL and AR-V7 in the indicated cells. **(B)** A natural product library was used to screen the inhibitors of AR-V7. **(C)** The structural diagram of Rutaecarpine (Rut). **(D)** Immunoblot analysis of AR-FL and AR-V7 in 22Rv1 cells exposed to Rut for 24 h or exposed to Rut (5 μM) for different lengths of time. **(E)** RT-PCR analysis of AR-V7 in 22Rv1 cells exposed to Rut for 12 h. Three independent experiments were performed. **(F)** Immunoblot analysis of AR-V7 protein level in 22Rv1 cells treated with Rut (5 μM) or vehicle for 12 h, and then exposed to cycloheximide (CHX) for different lengths of time. **(G)** Quantitative data of **(F)** are shown. Mean ± SD (n = 3). *P < 0.05. **(H)** Co-IP assay was performed using AR-V7 antibody or control IgG beads and immunoblotted for K48-Ub and AR-V7 in 22Rv1 treated with Rut for 12 h, and exposed to MG132(10 μM) for 6 h before harvest. **(I)** Quantitative data of **(H)** are shown. **(J)** Immunoblot analysis of AR-V7 in 22Rv1 cells exposed to Rut with or without Bortezomib (BTZ) for 12 h. **(K)** Quantitative data of **(J)** are shown. Mean ± SD (n = 3). *P < 0.05, ** P < 0.01, ^#^* P* < 0.05 versus BTZ (-), Rut (-),^ ###^* P* < 0.001 versus BTZ (-), Rut (-).

We first determined the expression of AR-FL and AR-V7 in various human PC cell lines and a prostatic stromal myofibroblast cell line WPMY-1. Our western blot results showed that AR-FL and AR-V7 were expressed in 22Rv1, LNCaP, and C4-2, but not in WPMY-1, PC3, and DU145 cells. Compared to LNCaP and C4-2 cell lines, the 22Rv1 cell line had the highest level of AR-V7 (Figure 1A). To identify a potential AR-V7 inhibitor, a natural product library containing 113 kinds of nature products (e.g., flavonoids, alkaloids, phenols, anthraquinones, quinones, and terpenes) was used to screen out the inhibitor of AR-V7 in 22Rv1 cells (Figure 1B). Among them, Rut, which is extracted from the dried fruit of *Evodia rutaecarpa* 22, exhibited the strongest inhibition on AR-V7 expression. The chemical structure of Rut was shown in a previous study (Figure 1C) 23. Western blot assay further observed that Rut dose-dependently down-regulated AR-V7 protein expression.

In contrast, Rut failed to decrease the protein level of AR-FL, indicating a special role of Rut in the regulation of AR-V7 expression. The time-chasing experiments also confirmed that Rut suppressed AR-V7 protein expression after 12 h (Figure 1D). The immunofluorescence results further demonstrated that Rut reduced the overall expression of AR-V7 in the cell (Figure S1A-B). These results suggest that Rut selectively down-regulates AR-V7.

We next determined whether Rut decreases the transcription of AR-V7 or promotes its degradation. Our Q-PCR results showed that Rut did not decrease the mRNA level of AR-V7 from 2.5 to 10 μmol/L (Figure 1E), while the cycloheximide (CHX)-chasing experiments showed that Rut shortened the half-life of AR-V7 protein (Figure 1F-G), suggesting that Rut promotes AR-V7 degradation. Our Co-IP assay further confirmed that Rut increased the Lys(K)48-linked ubiquitination of AR-V7 (Figure 1H-I). Moreover, the 20S proteasome inhibitor, bortezomib, notably reversed the Rut-induced AR-V7 protein down-regulation (Figure 1J-K), suggesting that Rut induced a proteasome-mediated degradation of AR-V7. These results suggest that Rut selectively promotes the K48-linked ubiquitination and proteasome-mediated AR-V7 degradation.

Previous studies have shown that Rut is an inhibitor of cyclooxygenase-2 (COX-2) 24, 25. To determine whether the Rut-induced K48-linked ubiquitination of AR-V7 was associated with its COX-2 inhibitory activity, we used parecoxib, another COX-2 inhibitor. Unlike Rut, treatment with parecoxib decreased the protein levels of both AR-V7 and AR-FL in 22Rv1, LNCaP, and C4-2 cells (Figure S2A). Additionally, parecoxib exhibited a similar inhibitory effect on the cell proliferation among 22Rv1, LNCaP, and C4-2 cell lines, which had a notable difference in the protein level of AR-V7 (Figure S2B-C). Unlike Rut, parecoxib at lower concentrations failed to affect the expression of AR-V7 and AR-FL (Figure S2D). More importantly, the knockdown of COX-2 using siRNA did not affect the protein level of AR-V7 and cell viability of PC cell lines (Figure S2E-F). Together, these findings demonstrate that COX-2 inhibition is not required for Rut-induced K48-linked ubiquitination of AR-V7.

### Rutaecarpine enhances the interaction between GRP78 and AR-V7

**Figure 2:**
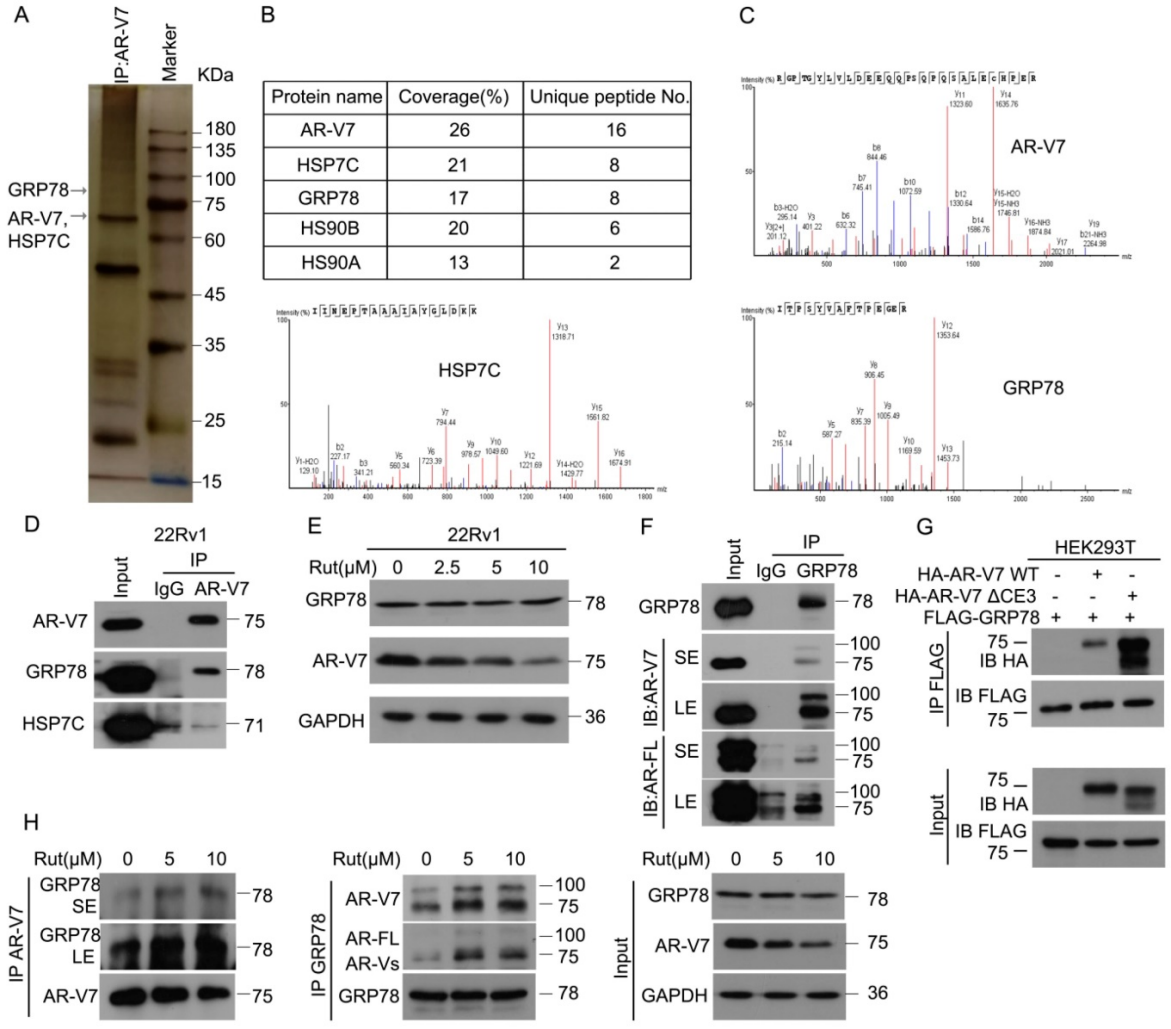
** Rutaecarpine increased the interaction between AR-V7 and GRP78. (A)** Co-IP assay was performed using AR-V7 antibody beads and subjected to Biological Mass Spectrometry in 22Rv1 cells. **(B)** AR-V7 interacting proteins are shown. **(C)** Ambipolar ion peaks of AR-V7, HSP7C and GRP78 are shown. **(D)** Co-IP assay was performed using AR-V7 antibody or control IgG beads and immunoblotted for GRP78 or HSP7C and AR-V7. **(E)** Western blot assay to detect the expression of HSP7C, GRP78 and AR-V7 in 22Rv1 cells exposed to Rut for 24 h. **(F)** Co-IP assay was performed using GRP78 antibody beads and immunoblotted for AR-FL and AR-V7 in 22Rv1 cells. SE, short exposure; LE, long exposure. **(G)** Co-IP assay was performed using FLAG-tag antibodies and immunoblotted for HA and FLAG in HEK293T cells transfected with FLAG-GRP78 and HA-AR-V7 or HA-AR-V7ΔCE3 for 48 h. **(H)** Co-IP assay was performed to determine the interaction of AR-V7/GRP78 in 22Rv1 cells exposed to Rut for 12 h.

To explore the underlying molecular mechanism of Rut-induced AR-V7 degradation, Co-IP combined with biomass spectrum assay was performed to identify the AR-V7 interacting proteins. The purified proteins from Co-IP using anti-AR-V7 antibodies were separated by SDS-PAGE, followed by silver staining (Figure 2A). Biomass spectrum analysis showed that AR-V7 interacted with several chaperones, including HSP7C (heat shock cognate 71 kDa protein), GRP78, HS90B (Hsp90-beta), HS90A (Hsp90-alpha) (Figure 2B-C). Indeed, molecular chaperones, such as HSP40, HSP70, and HSP90, are critical to the ubiquitin-mediated degradation of AR in certain PC cells and are proposed as anti-PC targets 26-28. We therefore wonder whether the chaperone machinery similarly controls the protein quality of AR-V7. Co-IP assay demonstrated that GRP78, but not HSP7C, strongly interacted with AR-V7 (Figure 2D), indicating that GRP78 may be the molecular chaperone controlling the protein quality of AR-V7. Moreover, GRP78 was more inclined to bind AR-V7 (~75KDa) than AR-FL (~100KDa) (Figure 2F), indicating that GRP78 preferentially interacts with AR-V7 in prostate cancer cells.

We wondered whether this selective interaction was mediated by the specific C-terminal (CE3) of AR-V7, a deletion mutant of CE3 (HA-AR-V7 ΔCE3) and a wide-type HA-AR-V7 (HA-AR-V7 WT) plasmid were next engineered and co-transfected with FLAG-GRP78 into HEK293T cells. However, AR-V7 Δ CE3 more strongly interacted with GRP78 than HA-AR-V7 WT (Figure 2G), suggesting that the specific CE3 of AR-V7 is not required for its binding to GRP78, and this selective interaction may be influenced by the different steric configurations of AR-V7 and AR-FL. We further determined whether Rut changed the expression of AR-V7 or the protein interaction between AR-V7 and GRP78. Our western blot and Co-IP results showed that Rut did not notably affect the protein level of GRP78, but unexpectedly increased the interaction between GRP78 and AR-V7 and AR-FL (Figure 2E, H). Collectively, these results demonstrate that GRP78 preferentially binds to AR-V7 and this interaction further increased following Rut treatment.

### GRP78 mediates rutaecarpine-induced AR-V7 degradation

**Figure 3:**
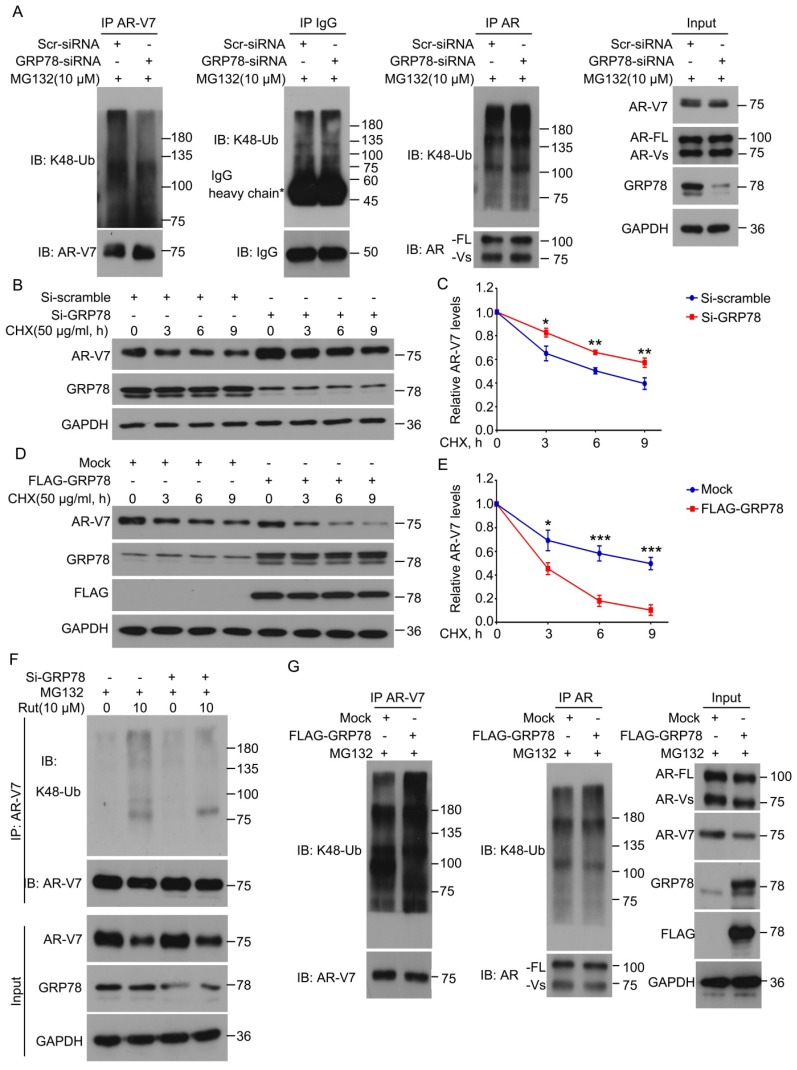
** GRP78 mediated the degradation of AR-V7 induced by rutaecarpine. (A)** Co-IP assay was performed using AR-V7, AR or IgG control antibody beads and immunoblotted for K48-Ub and AR-V7, AR, or IgG control in 22Rv1 treated with GRP78 siRNAs or control siRNAs for 48 h, and exposed to MG132 (10 μM) for 6 h before harvest. **(B)** Immunoblot analysis of AR-V7 in 22Rv1 cells treated with GRP78 siRNA (50 nM) or control siRNA for 48 h, and exposed to CHX for different lengths of time. **(C)** Quantitative data of **(B)** are shown. Mean ± SD (n = 3). *P < 0.05, ** P < 0.01. **(D)** Immunoblot analysis of AR-V7 in 22Rv1 cells treated with FLAG-GRP78 plasmid or control plasmid for 48 h, and then treated with CHX for different lengths of time.** (E)** Quantitative data of **(D)** are shown. Mean ± SD (n = 3). *P < 0.05, *** P < 0.001.** (F)** Co-IP assay was performed to detect the K48-linked ubiquitination levels of AR-V7 in 22Rv1 cells exposed to Rut (10 μM) for 12 h, with or without GRP78 siRNAs treatment for 48 h, and exposed to MG132 (10 μM) for 6 h before harvest. **(G)** Co-IP assay was used to detect the K48-linked ubiquitination levels of AR-V7 and AR in 22Rv1 cells transfected with FLAG-GRP78 for 48 h, and exposed to MG132 (10 μM) for 6 h before harvest.

Because GRP78 interacts with AR-V7, we next determined whether GRP78 mediates the ubiquitination and degradation of AR-V7. The knockdown of GRP78 by siRNA significantly decreased the abundance of K48-linked ubiquitination of AR-V7 and moderately increased the protein level of AR-V7 (Figure 3A). However, the knockdown of GRP78 did not affect the abundance of K48-linked ubiquitination of AR-FL as well as the protein level of AR-FL (Figure 3A). These findings indicate that GRP78 may selectively mediate the ubiquitination of AR-V7, but not AR-FL.

To determine whether GRP78 affects the half-life of AR-V7, the CHX-chasing experiments were performed in further investigations. These experiments showed that the knockdown of GRP78 notably prolonged the half-life of AR-V7 protein (Figure 3B-C). Moreover, the forced-expression of GRP78 remarkably shortened the half-life of AR-V7 and increased the expression of AR-V7 (Figure 3D-E). Additionally, the overexpression of GRP78 further increased the abundance of the K48-linked ubiquitination of AR-V7, but not AR-FL (Figure 3G). These results suggest that GRP78 physically interacts with AR-V7 and then mediates the K48-linked ubiquitination and degradation of AR-V7 in PC cells.

To further investigate whether GRP78 mediates Rut-induced ubiquitination of AR-V7, Co-IP and western blot assays were performed in 22Rv1 cells treated with Rut with or without knockdown of GRP78. The results showed that the knockdown of GRP78 not only reversed the Rut-induced downregulation of AR-V7, but also rescued the Rut-induced K48-linked ubiquitination of AR-V7 (Figure 3F). These findings suggest that GRP78 is a key regulator of Rut-induced K48-linked ubiquitination and degradation of AR-V7.

### GRP78 recruits the E3 ligase SIAH2 to degrade AR-V7

**Figure 4:**
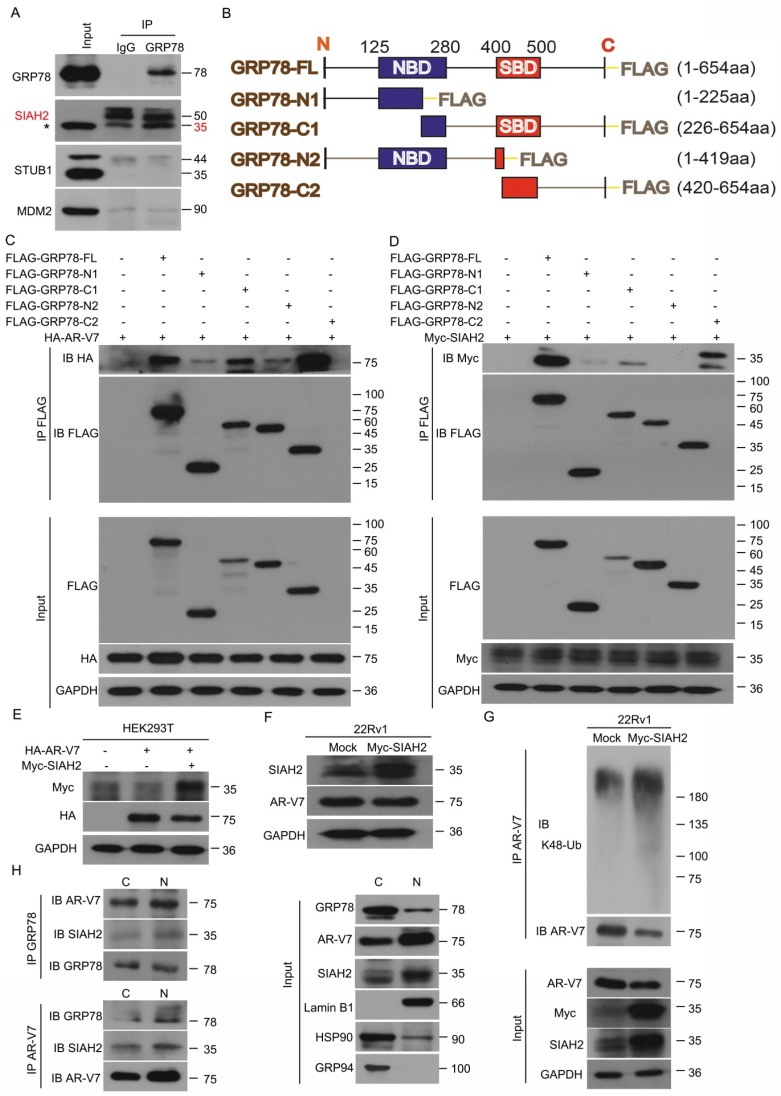
** GRP78 recruits SIAH2 to mediate the degradation of AR-V7. (A)** Co-IP assay was performed to identify the interaction between GRP78 and SIAH2, STUB1 or MDM2 in 22Rv1 cells. **(B)** Truncated mutants of GRP78 fused with FLAG-tag on their C-terminals were engineered and **(C)** co-transfected with HA-AR-V7 into HEK293T cells. Co-IP assays were performed using FLAG-tag antibodies and immunoblotted for HA and FLAG. **(D)** Truncated mutants of GRP78 fused with FLAG-tag on their C-terminals were co-transfected into HEK293T cells. Co-IP assays were performed using Flag-tag antibodies and immunoblotted for Myc and FLAG. IgG was blocked using mouse anti-rabbit IgG antibodies (Cell Signaling Technology, #5127) in the following immunoblots. **(E)** Western blot assays were performed using Myc or HA antibodies in HEK293T cells transfected with HA-AR-V7 plasmids with or without Myc-SIAH2 plasmids for 48 h. **(F)** Western blot assays were performed using SIAH2 or AR-V7 antibodies in 22Rv1 cells transfected with Myc-SIAH2 plasmids for 48 h. **(G)** Co-IP assay was performed using AR-V7 antibodies and immunoblotted for K48-Ub and AR-V7 in 22Rv1 cells transfected with Myc-SIAH2 plasmids for 48 h, and exposed to MG132 (10 μM) for 6 h before harvest. **(H)** Cytoplasmic and nuclear proteins were separated from 22Rv1 cells. Co-IP assays were performed using AR-V7 antibodies or GRP78 antibodies and immunoblotted for GRP78, SIAH2 and AR-V7 in cytoplasmic and nuclear extracts.

Since GRP78 is a molecular chaperone, we investigated whether GRP78 interacts with E3 ligases to mediate the ubiquitination and degradation of AR-V7. Early studies have shown that certain E3 ligases, such as MDM2, SIAH2, and STUB1/CHIP, interact with AR-FL to mediate its ubiquitination 29-32. Our Co-IP and western blot assays found that GRP78 interacted with SIAH2, but not MDM2 and STUB1 (Figure 4A), indicating a potential role of SIAH2 in the regulation of AR-V7 degradation. Indeed, the overexpression of GRP78 increased the binding between AR-V7 and SIAH2 (Figure S3A). These findings suggest that SIAH2 may be a E3 ligase responsible for AR-V7 degradation.

To further determine the binding domain of GRP78 to AR-V7 or SIAH2, several truncated mutants of FLAG-GRP78 were engineered and transfected with HA-AR-V7 or Myc-SIAH2 into HEK293T cells (Figure 4B). This assay showed that the C-terminal of GRP78, which contained a substrate binding domain, was essential for the binding of GRP78 to AR-V7 and SIAH2 (Figure 4C-D). Notably, the forced-expression of SIAH2 decreased the expression of both exogenous and endogenous AR-V7 in HEK293T cells and 22Rv1 cells, respectively (Figure 4E-F). Moreover, the forced-expression of SIAH2 increased the abundance of K48-linked ubiquitination of AR-V7 (Figure 4G), indicating that SIAH2 is an E3 ligase responsible for the ubiquitination and degradation of AR-V7.

We next assayed the subcellular location of the GRP78-AR-V7-SIAH2 protein complex in PC cells. Immunofluorescence assays were performed in 22Rv1 cells that transfected with HA-AR-V7 or FLAG-GRP78. GRP78 highly presented in the cytoplasm, but moderately located in the nucleus. SIAH2 was expressed in both the cytoplasm and the nucleus (Figure S3B). AR-V7 was highly found in the nucleus, but moderately located in the cytoplasm (Figure S3C-D). These image assays indicate the protein complex between AR-V7, GRP78, and SIAH2 exists in both the cytoplasm and the nucleus.

To further confirm the location of the GRP78-AR-V7-SIAH2 protein complex in 22Rv1 cells, Co-IP assay was performed using the nuclear or cytoplasmic extracts. The GRP78-AR-V7-SIAH2 protein complexes were more abundant in the nucleus than in the cytoplasm (Figure 4H). Furthermore, the treatment of Rut reduced the expression of nuclear as well as cytoplasmic AR-V7, and decreased the expression of nuclear GRP78 and SIAH2 in 22Rv1 cells (Figure S3E). These findings indicate that the GRP78-AR-V7-SIAH2 protein complex may form in the nucleus and then export to the cytoplasm for the proteasome-mediated degradation of AR-V7.

### Rutaecarpine suppresses the proliferation of CRPC cells *in vitro*

**Figure 5:**
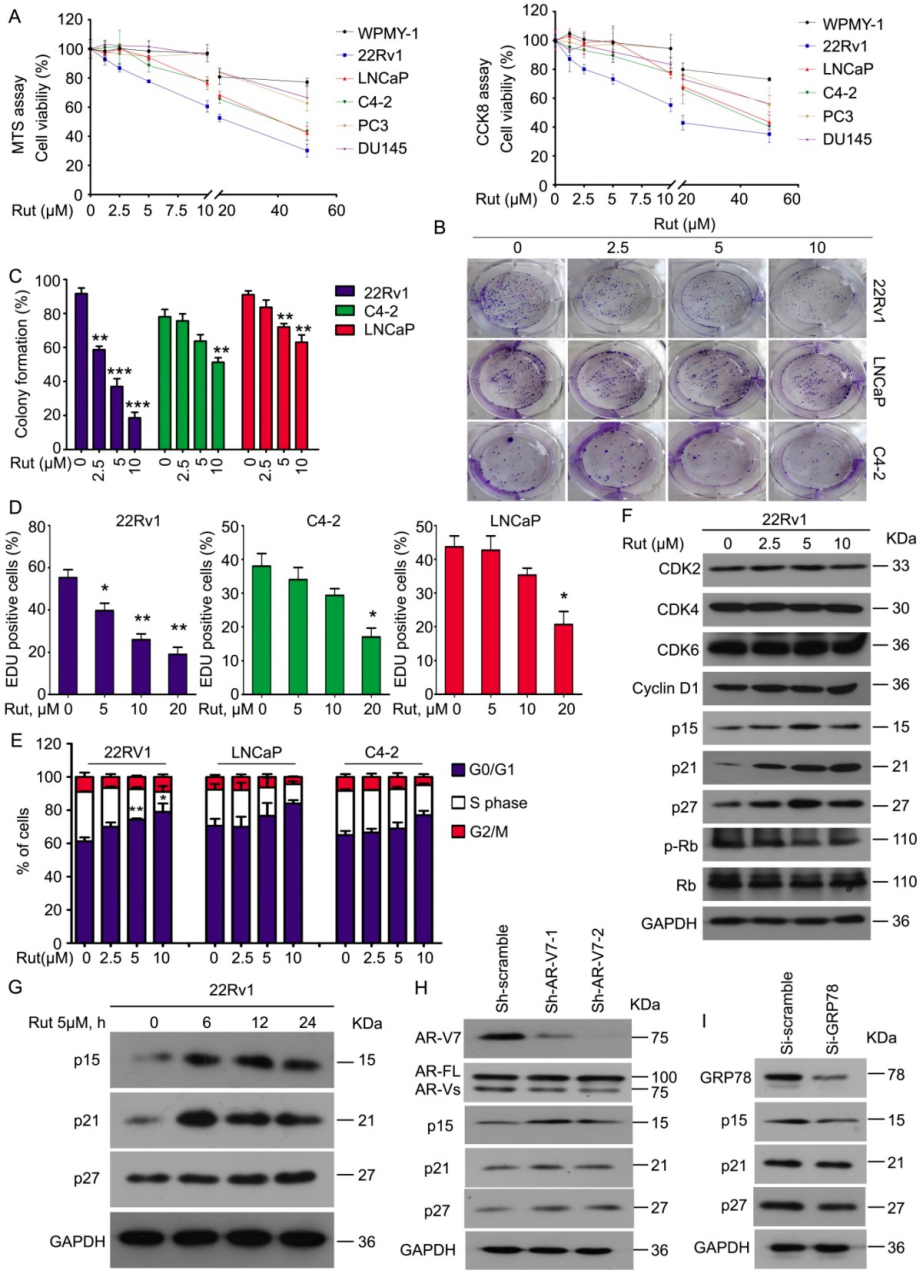
** Rutaecarpine suppressed PC growth in an AR-V7 expression-dependent manner. (A)** MTS and CCK8 assays were performed in WPMY-1, 22Rv1, LNCaP, C4-2, PC3 and DU145 cells exposed to Rut for 48 h. **(B)** A colony formation assay was performed in 22Rv1, LNCaP and C4-2 cells exposed to Rut for 2 weeks. **(C)** Quantitative data of **(B)** are shown. Mean ± SD (n = 3). **P<0.01, ***P<0.001. **(D)** An EDU staining assay was performed in 22Rv1, LNCaP and C4-2 cells exposed to Rut for 48 h. Quantitative data are shown. Mean ± SD (n = 3). *P<0.05, **P<0.01. **(E)** A fluorescence-activated cell sorting analysis (FACS) was performed in 22Rv1, LNCaP and C4-2 cells exposed to Rut for 24 h. Quantitative data of three independent experiments are shown. *P<0.05, **P<0.01. **(F)** Immunoblot analysis of CDK2/4/6, cyclin D1, p15, p21, p27, p-Rb, and Rb in 22Rv1 cells exposed to Rut for 24 h. **(G)** Immunoblot analysis of p15, p21, and p27 in 22Rv1 cells exposed to Rut for various lengths of time. **(H)** Immunoblot analysis of p15, p21, and p27 in 22Rv1 cells stably expressing control shRNAs or AR-V7 shRNAs. **(I)** Immunoblot analysis of p15, p21, and p27 in 22Rv1 cells exposed to GRP78 or control siRNAs.

To determine whether Rut selectively overcomes AR-V7 positive prostate cancer cells *in vitro*, a cell viability analysis was performed using MTS and CCK8 assays in normal prostate stromal (WPMY-1), AR-V7-positive (22Rv1, LNCaP, and C4-2), and AR-V7-negative (PC3 and DU145) cell lines following Rut treatment. Rut dose-dependently decreased the viability of AR-V7-positive cells, especially in 22Rv1 cells, which have the highest expression level of AR-V7. Meanwhile, normal prostate stromal (WPMY-1) and AR-V7-negative (PC3 and DU145) cell lines were not sensitive to Rut treatment at the concentration of 0~20 μM (Figure 5A). Colony formation experiments and EDU staining assays fìurther showed that Rut decreased the cell proliferation of 22Rv1, LNCaP, and C4-2 cells, especially in 22Rv1 cells (Figure 5B-D). These findings suggest that Rut exhibits an AR-V7-dependent anti-CRPC activity.

To further investigate whether Rut affects the cell cycle progression of PC cells, PI staining and flowcytometry were used to detect the cell cycle distribution. Consistent with the cell viability and colony formation assays, Rut also caused cell cycle arrest in the G0/G1 phase in these CRPC cells, especially in 22Rv1 cells (Figure 5E). Western blot analysis further demonstrated that Rut did not affect the expression of CDK2, CDK4, CDK6, and cyclin D1, which are the promoters of the G0/G1 to S phase transition. In contrast, Rut increased the expression of p15, p21, and p27, which are suppressors of the G0/G1 to S phase transition, in a dose- and time-dependent manner (Figure 5F-G). These findings indicate that Rut may induce G0/G1 arrest through up-regulating the protein level of p15, p21, and p27, but not CDK family members. Importantly, the knockdown of AR-V7 increased (Figure 5H), whereas the knockdown of GRP78 (Figure 5I) decreased the expression of p15, p21, and p27. Additionally, the forced expression of GRP78 increased the expression of these molecules (Figure S4A). These RNAi studies further suggest that GRP78-mediated AR-V7 degradation is responsible for cell cycle arrest in CRPC cells.

To further address the relationship between Rut treatment and AR-V7 degradation in the regulation of cell growth of CRPC cells, we performed the AR-V7 rescue experiments using lentiviruses containing AR-V7 plasmids. Indeed, the overexpression of AR-V7 significantly reversed Rut-induced inhibition of cell viability and colony formation (Figure S4B-C). These findings suggest that the anti-CRPC activity of Rut depends on AR-V7 degradation.

### Rutaecarpine suppresses the tumor growth of CRPC cells *in vivo*

**Figure 6:**
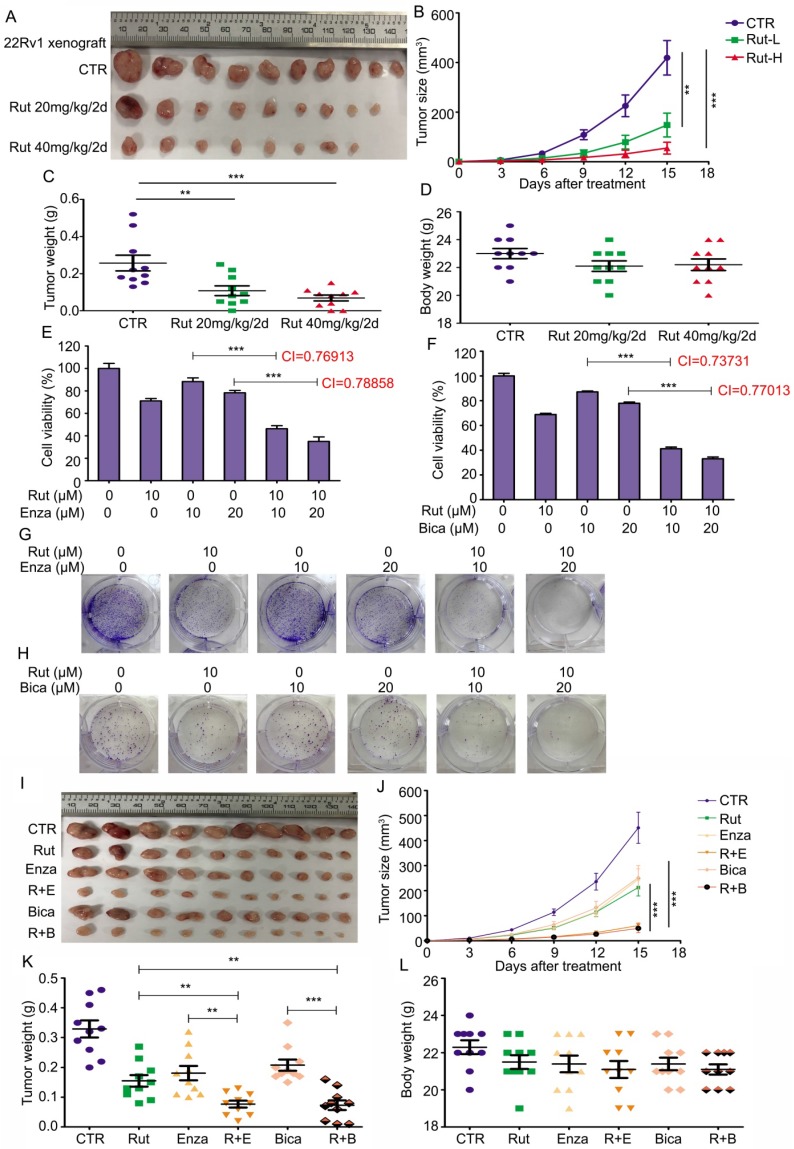
** Rutaecarpine suppressed the growth of CRPC in *vivo* and enhanced the sensitivity of CRPC cells to anti-androgen therapeutics. (A)** 22Rv1 xenografts were established on BALB/c nude mice and randomly separated into 3 groups, and treated with Rut 20mg/kg/2d (i.p.), 40mg/kg/2d (i.p.), or vehicle for 15 days. Xenograft images, **(B)** tumor size, **(C)** tumor weight, and **(D)** body weight of nude mice are shown. Mean ± SD (n = 10). **P<0.01, ***P<0.001. **(E)** and **(F)** MTS assay was performed in 22Rv1 cells exposed to Enza/Bica with or without Rut for 48 h. Mean ± SD (n = 3). ***P<0.001. CI, combination index, was calculated from three independent experiments. **(G)** and **(H)** Colony formation assay was performed in 22Rv1 cells exposed to Enza/Bica with or without Rut for 2 weeks. **(I)** 22Rv1 xenografts were established on BALB/c nude mice and randomly separated into 6 groups, and treated with vehicle, Rut 20mg/kg/2d (i.p.), Enza 25mg/kg/2d (p.o.), Bica 25mg/kg/2d (p.o.), Rut+Enza, or Rut+Bica for 15 days. Xenograft images, **(J)** tumor size, **(K)** tumor weight, and **(L)** body weight of nude mice are shown. Mean ± SD (n = 10). **P<0.01, ***P<0.001.

To determine the anticancer activity of Rut *in vivo*, 22Rv1 xenograft models were established. The pharmacokinetic studies of Rut on mice were reported as previously 33-36. Rut notably suppressed the growth of CRPC, and three xenografts were diminished after Rut treatment (Figure 6A). The tumor sizes and tumor weights in Rut-treated groups were significantly reduced (Figure 6B-C), suggesting that Rut is effective in xenograft models in nude mice. As expected, the immunohistochemistry results showed that Rut remarkably decreased the protein levels of Ki67 and AR-V7 on the xenograft tissues (Figure S5A-D). In addition, Rut slightly, but not significantly, reduced the body weights of nude mice (Figure 6D). Moreover, Rut did not lead to tissue (e.g., liver and kidney) injury in nude mice at the concentrations of 20 mg/kg/2d or 40 mg/kg/2d, indicating that the toxic side effect of Rut was relatively low (Figure S5E). Together, these findings indicate that Rut has a predominantly anti-CRPC activity both *in vitro* and *in vivo*.

Given that AR-V7 is a key player in mediating the resistance to antiandrogen therapies, we asked whether Rut could enhance the sensitivity of CRPC cells to antiandrogen agents, including Enza and bicalutamide (Bica). To address this question, a cell viability assay was first performed and the combination index (CI) values were calculated in 22Rv1 cells exposed to Rut and Enza or Bica. The results showed that Rut synergistically enhanced the sensitivity of CRPC cells to Enza and Bica (CI<1 represents a synergistic effect for two chemicals) (Figure 6E-F). The colony formation results also confirmed that the proliferation inhibitory effects in the combined treatment groups were more efficacious than the single (Rut, Enza, or Bica) treatment groups (Figure 6G-H; Figure S6A-B). Additionally, the forced expression of GRP78 similarly increased the sensitivity of 22Rv1 cells to Enza and Bica (Figure S7A-B), suggesting that the reduction of AR-V7 by Rut and GRP78 is a reliable way to overcome castration resistance.

To further evaluate the combined effects of Rut + Enza / Bica *in vivo*, the 22Rv1 xenograft models were established and randomly separated as six groups, and treated with Rut, Enza, Bica alone or their combination. The results showed that the reduction of tumor sizes and tumor weights were more remarkable in combination groups than in the single-treatment groups (Figure 6I-K), indicating that the combinations of the Rut and antiandrogen agents were effective in CRPC models *in vivo*. Of note, the reductions of body weight of mice between the combination groups and single-treatment groups were not significant (Figure 6L), indicating that the combined strategy of Rut and antiandrogen agents was safe.

Taken together, these findings suggest that Rut not only potently inhibited CRPC *in vivo*, but also enhanced the sensitivity of CRPC to antiandrogen therapies.

## Discussion

Currently, hormone therapy remains the major approach for managing patients with PC. Unfortunately, in 10-20% of patients, CRPC develops and that most patient with CRPC show an aberrant reactivation of AR signaling despite anti-androgen (or antiendocrine) treatment 37. Increasing studies have made efforts to overcome CRPC or resensitize CRPC cells to anti-androgen and docetaxel treatment by targeting a specific molecule 38, 39, that represent promising targets for PC therapy. Although the mechanisms underlying the AR reactivation in the development of CRPC are incompletely understood, AR-V7 has been well-demonstrated as the key factor contributing to the arousal of AR signaling and the progression of CRPC. In this study, we identified Rut as a novel chemical that selectively induces AR-V7 protein degradation via the activation of the GRP78-SIAH2 pathway.

Natural products are attractive sources for drug discovery 40 and have aroused numerous research interests for their advantages of low toxicity compared to standard chemotherapy 41. Although there are some technical barriers in screening natural products against a specific molecular target, the emergence of natural product libraries makes screening more convenient. The current screen study found that Rut was effective in selectively inhibiting AR-V7 compared to AR-FL**.** Rut, an alkaloid extracted from *Evodia rutaecarpa*, was proposed to exhibit bioactivity in cardiac hypertrophy prevention, adipogenesis/lipogenesis inhibition and atherosclerosis suppression 22, 42, 43. Rut exerts anti-inflammatory effects probably via inhibition of COX-2 24. However, our current study showed that the genetic and pharmacological activation of COX-2 did not exert paralleled effects in the downregulation of AR-V7 with Rut, indicating that Rut-induced AR-V7 degradation was independent of its effect of COX-2 inhibition. Our western blot analysis, CHX-chasing study, Co-IP assay, and bortezomib rescue experiments further demonstrated that Rut inhibited the AR-V7 signaling by selectively promoting its K48-linked ubiquitination for the proteasomal degradation.

Using Co-IP assays and biological mass spectrometry, various critical protein interacting partners of AR-V7 were identified in the current study. Because of the importance of molecular chaperones in the degradation of AR-FL, this study focused on the chaperones that may interact with AR-V7 and similarly influence its degradation. We confirmed that GRP78 strongly interacts with AR-V7, but weakly interacts with AR-FL. This observation was consistent with Rut down-regulating AR-V7, but not AR-FL in a certain range of doses. By using a specific AR-V7 C-terminal deletion mutant (AR△CE3), this study revealed that the CE3 was not required for binding AR-V7 to GRP78, suggesting that the difference of steric configuration between AR-V7 and AR-FL affected their binding capacities to GRP78. Interestingly, Rut increased the interaction between AR-V7 and GRP78 in CRPC cells. GRP78 is an endoplasmic reticulum (ER) chaperone that masters the unfolded protein response (UPR) which is just recently identified as an effective biomarker for treating hepatocellular cancer 44, 45. The UPR triggered by GRP78 is a well-characterized mechanism for tumor survival in many circumstances 44, yet an excessive ER stress may induce apoptosis in cancer cells 46. A recent study shows that AR-FL, mutant AR, and AR-V7 could be down-regulated by riluzole via ER stress and p62-mediated selective autophagy 47, providing an alternative mechanism for the degradation of AR and AR-Vs. The current study hypothesized that GRP78 is a critical mediator of the K48-linked ubiquitination and degradation of AR-V7 due to the following evidence: 1) the knockdown of GRP78 decreased the abundance of K48-linked ubiquitination of AR-V7 and prolonged the half-life of AR-V7; and 2) the overexpression of GRP78 increased the abundance of K48-linked ubiquitination of AR-V7 and shortened the half-life of AR-V7. Moreover, the knockdown of GRP78 reversed the Rut-induced K48-linked ubiquitination and the downregulation of AR-V7. These findings strongly demonstrated that GRP78 mediated Rut-induced AR-V7 degradation.

Chaperones are able to recruit E3 ligases to mediate the ubiquitination of their substrates 48-51. These findings enforce the notion that the function of chaperones is not only to promote protein folding and maturation, but also to mediate protein degradation. In this study, GRP78 also shared a similar capacity because it recruits the E3 ligase SIAH2 to mediate the ubiquitination and degradation of AR-V7 in CRPC cells. Although SIAH2 was proposed as a tumor promoter that mediates the K63-linked ubiquitination and nuclear translocation of AR-FL 31, its role in the regulation of AR-V7 or other AR-Vs and the progression of CRPC remains largely unknown. An interesting phenomenon is that a GRP78-AR-V7-SIAH2 degraded complex might form in the nucleus and subsequently export to the cytoplasm for proteasome anchoring because the GRP78-AR-V7-SIAH2 complexes were more abundant in the nucleus than in the cytoplasm. It is worth noting that the C-terminal of GRP78 is the essential domain of its binding to AR-V7 and SIAH2. In this complex, GRP78 may work as a molecular platform for the binding of AR-V7 and SIAH2, thereby increasing their affinity and facilitating the ubiquitination of AR-V7 because the overexpression of GRP78 increased the interaction of AR-V7 and SIAH2 and the K48-linked ubiquitination and degradation of AR-V7. More importantly, Rut increased affinity between GRP78 and AR-V7, and formation of the GRP78-AR-V7-SIAH2 complex, thereby promoted the degradation of AR-V7, indicating that Rut exerts this pharmacological action in the nucleus of CRPC cells. This study unraveled a unique pathway for the degradation of AR-V7 and may provide a druggable target for overcoming CRPC by targeting the GRP78-AR-V7-SIAH2 complex.

By confirming that Rut enhanced formation of the GRP78-AR-V7-SIAH2 degraded complex and thereby promoted the degradation of AR-V7, we further explored its anti-CRPC effects *in vitro* and *in vivo*. Interestingly, our cell viability assay, clonogenic assay, and EDU staining assay showed that Rut suppressed the proliferation of PC cells in a different manner. These effects were more visible in AR-V7-overexpressed 22Rv1 cells, compared to AR-FL-expressed or AR-V7-deficient cells. These findings were consistent with the previous finding that Rut selectively down-regulates AR-V7. Further explorations showed that Rut arrested the G0/G1 phase of the cell cycle via the upregulation of the expression of inhibitors of the G0/G1 phase to S phase, including p15, p21, and p27, promoted by the increased formation of GRP78/SIAH2/AR-V7 complexes in the nucleus. Additionally, the knockdown of AR-V7 or overexpression of GRP78 increased the expression of p15, p21, and p27, while the knockdown of GRP78 decreased their expression. The restoration of AR-V7 rescued the Rut-induced cell growth suppression *in vitro*. Rut, exhibiting low cytotoxicity, also dramatically suppressed the tumor growth of CRPC models *in vivo*. Moreover, Rut could enhance the sensitivity of CRPC models to antiandrogen agents, including Enza and Bica, indicating that the use of Rut in CRPC may possess great translational value.

**Figure 7:**
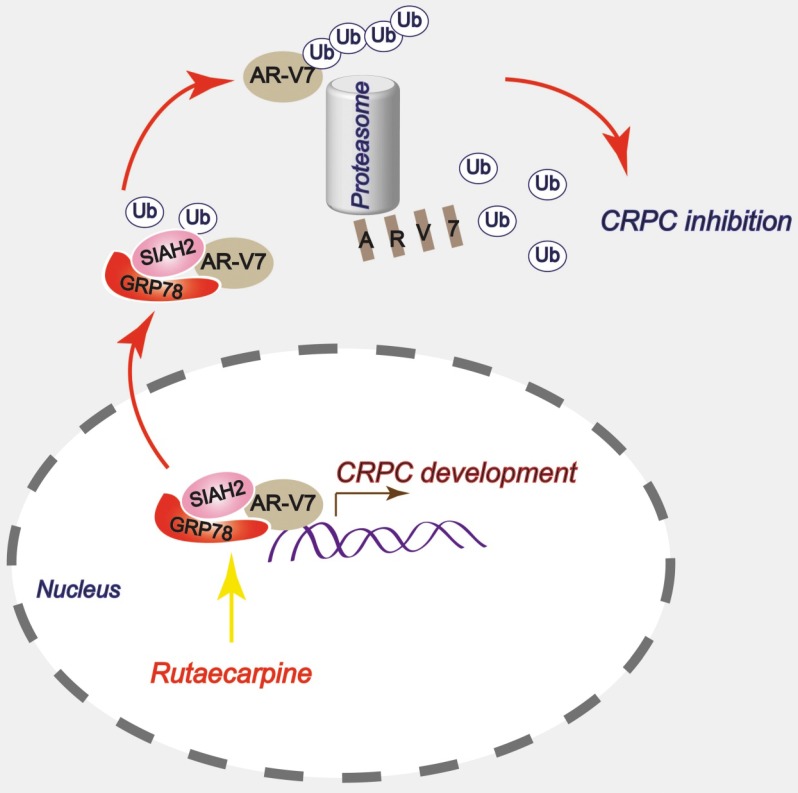
A proposed model by which Rutaecarpine inhibited AR-V7 and CRPC.

In summary, this study has provided a novel anti-CRPC strategy by targeting AR-V7 with Rut *in vitro* and *in vivo*. Mechanically, this effect relies on the Rut-induced formation of the GRP78-AR-V7 protein complex, which further recruits the E3 ligase SIAH2 to directly promote the ubiquitination of AR-V7 (Figure 7).

## Supplementary Material

Supplementary figures.
