# Supplementary material for: Targeting GRP78-dependent AR-V7 protein degradation overcomes castration-resistance in prostate cancer therapy
Source: Theranostics. 2020 Feb 10;10(8):3366–81. doi: 10.7150/thno.41849 (PMC7069092; doi:10.7150/thno.41849)
Supplement: Supplementary file 1 — Supplementary figures. [file thnov10p3366s1.pdf]

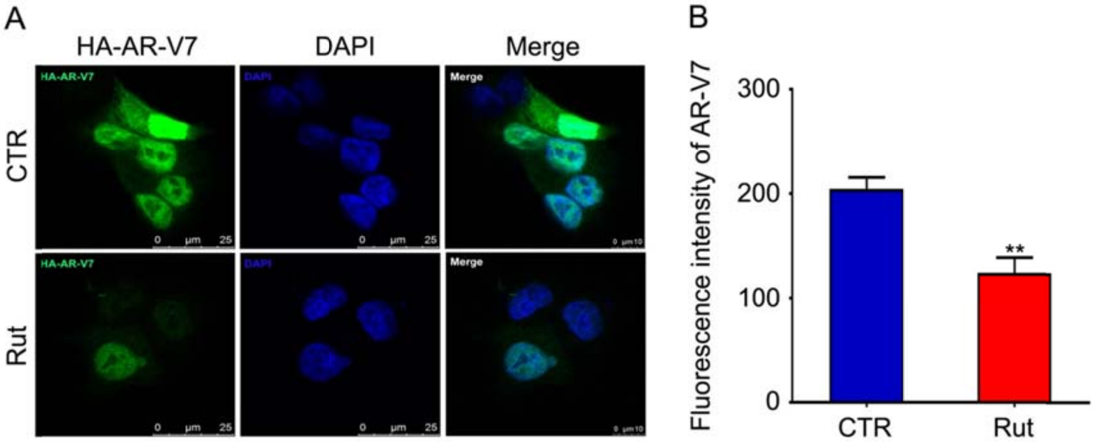

Figure S1. Rutaecarpine stood out as a selective inhibitor of AR-V7 by promoting its degradation in proteasome. (A) Confocal assay was performed using HA-tag antibodies in 22Rv1 cells transfected with HA-AR-V7 for 48 h, and subsequently exposed to Rut (10 μM) or vehicle for 12 h. Scale bars represent 25 μm or 10 μm. **(B)** Quantitative data of **(A)** is shown. Mean ± SD (n = 3). ** $P < 0.01$.

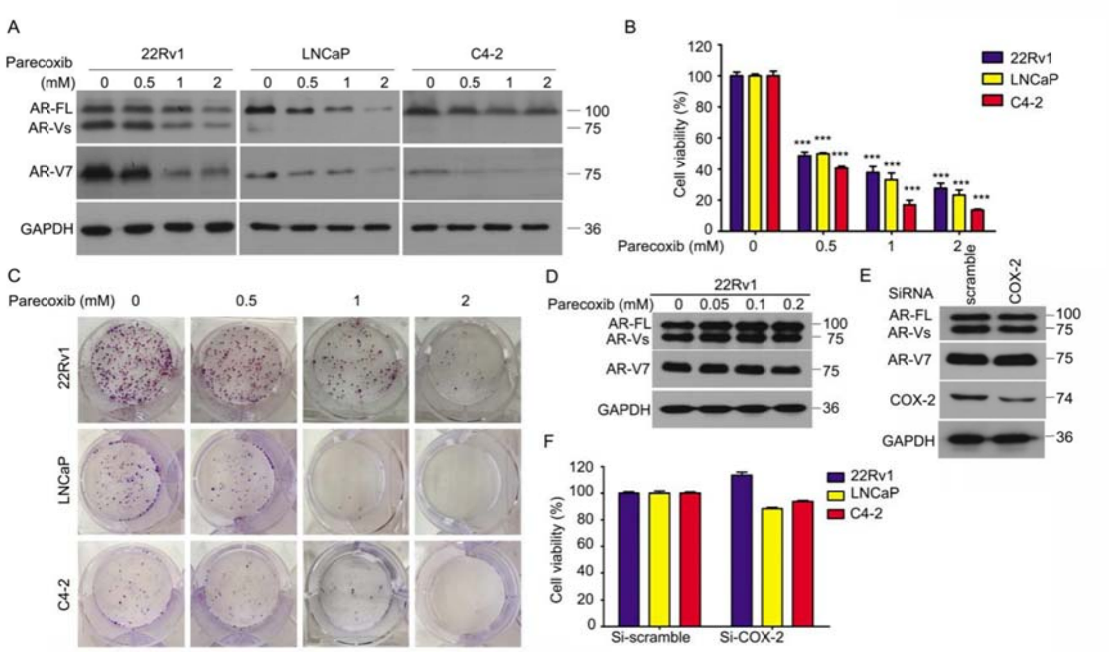

Figure S2. Rutaecarpine induced AR-V7 degradation was independent of its COX-2 inhibitory activity. (A) Immunoblot analysis of AR-FL and AR-V7 in 22Rv1, LNCaP and C4-2 cells exposed to parecoxib for 24 h. **(B)** MTS assay was performed

in 22Rv1, LNCaP and C4-2 cells exposed to parecoxib for 24 h. Mean ± SD (n = 3). ****P*<0.001. **(C)** Colony formation analysis of 22Rv1, LNCaP, and C4-2 cells exposed to parecoxib for 2 weeks. **(D)** Immunoblot analysis of AR-FL and AR-V7 in 22Rv1 cells exposed to the indicated doses of parecoxib for 24 h; and **(E)** COX-2 siRNAs for 48h. **(F)** MTS assay was performed in 22Rv1 exposed to COX-2 siRNAs for 48h. Mean ± SD (n = 3).

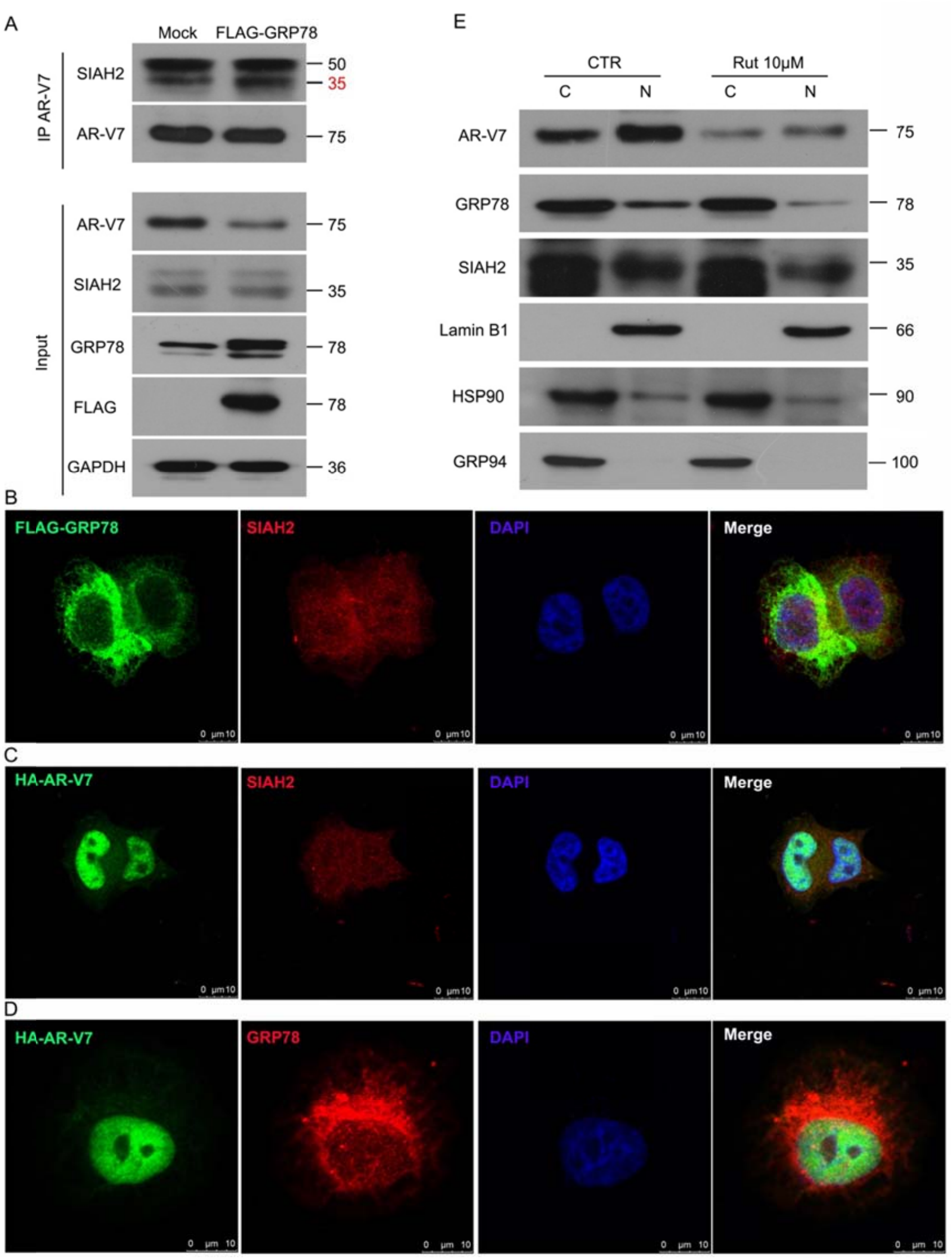

Figure S3. Overexpression of GRP78 increased the interaction between AR-V7 and SIAH2. (A) Co-IP and western blot assays were performed to detect the interaction between AR-V7 and SIAH2 in 22Rv1 cells treated with FLAG-GRP78 or control plasmid for 48 h. **(B)** Confocal assay was performed using FLAG-tag and

SIAH2 antibodies in 22Rv1 cells transfected with FLAG-GRP78 for 48 h. **(C)** Confocal assay was performed using HA-tag and SIAH2 antibodies in 22Rv1 cells transfected with HA-AR-V7 for 48 h. **(D)** Confocal assay was performed using FLAG-tag and GRP78 antibodies in 22Rv1 cells transfected with FLAG-GRP78 for 48 h. Scale bars represent 10 μm. **(E)** Immunoblot analysis of AR-V7, GRP78, and SIAH2 in cytoplasmic and nuclear extracts of 22Rv1 cells exposed to Rut for 12 h. HSP90 and lamin B1 were used as cytoplasmic and nuclear internal controls respectively. GRP94 was used as an endoplasmic reticulum (ER)-specific marker.

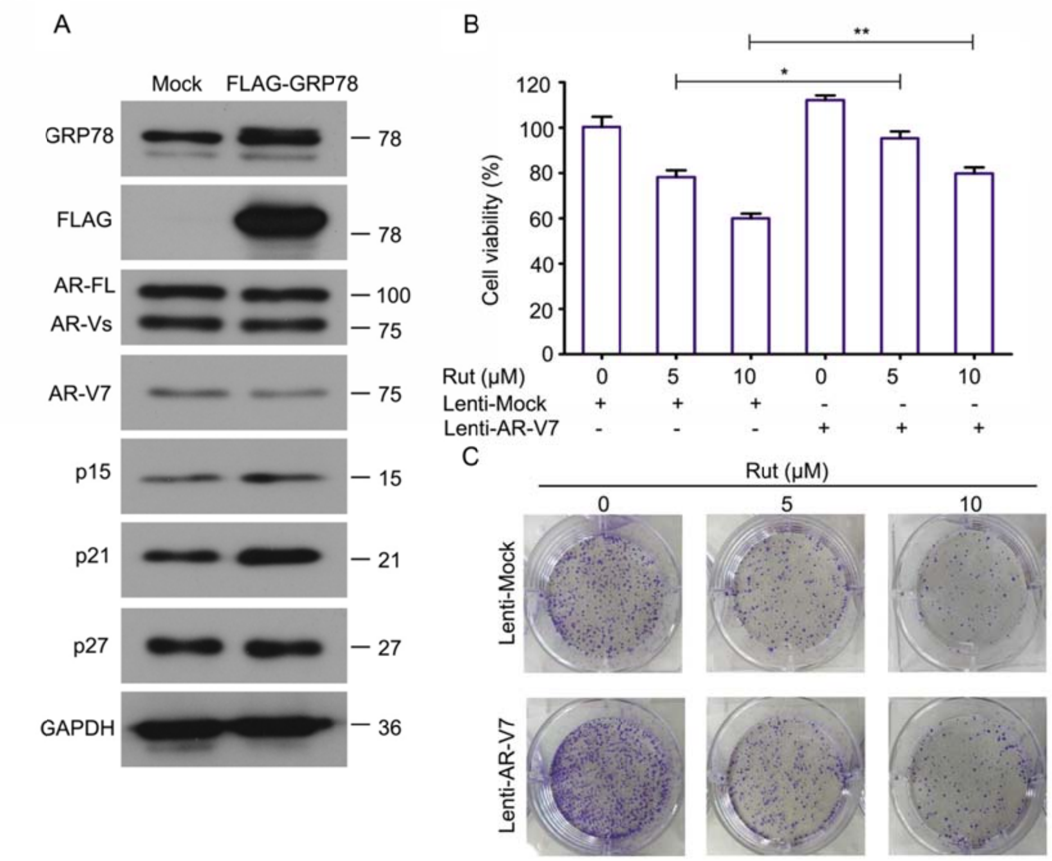

Figure S4. Forced expression of AR-V7 reversed the anti-CRPC effects of rutaecarpine. (A) Immunoblot analysis of GRP78, FLAG, AR-FL, AR-V7, p15, p21

and p27 in 22Rv1 cells treated with FLAG-GRP78 or control plasmid for 48 h. **(B)** Cell viability assays and **(C)** colony formation assays were performed in 22Rv1 cells transfected with lentiviruses containing recombinant AR-V7 or control plasmids for 48 h, and subsequently exposed to increasing concentrations of Rut for 48 h. **P*<0.05, ***P*<0.01.

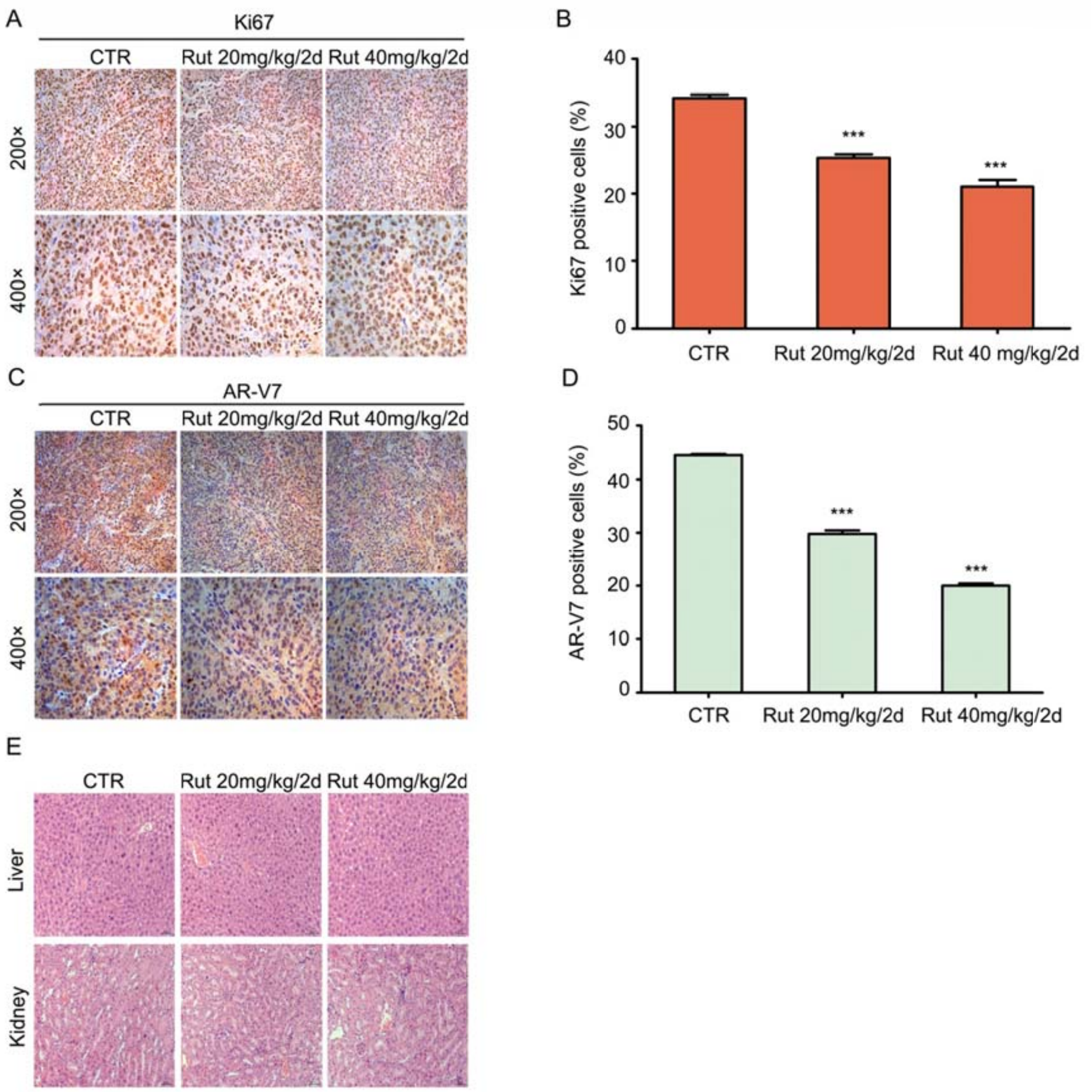

Figure S5. Rutaecarpine decreased the expression of AR-V7 and Ki67 *in vivo*. Immunohistochemistry assay was performed to determine the expression and localization of Ki67 and AR-V7 in 22Rv1 xenografts. **(A, C)** Representative images

in each group are shown at 200× and 400×. **(B, D)** Quantitative data of **(A, C)** are shown. Mean ± SD (n = 3). ***P<0.001. **(E)** H&E staining assay was performed in liver and kidney tissues of nude mice. Representative images in each group are shown at 200×.

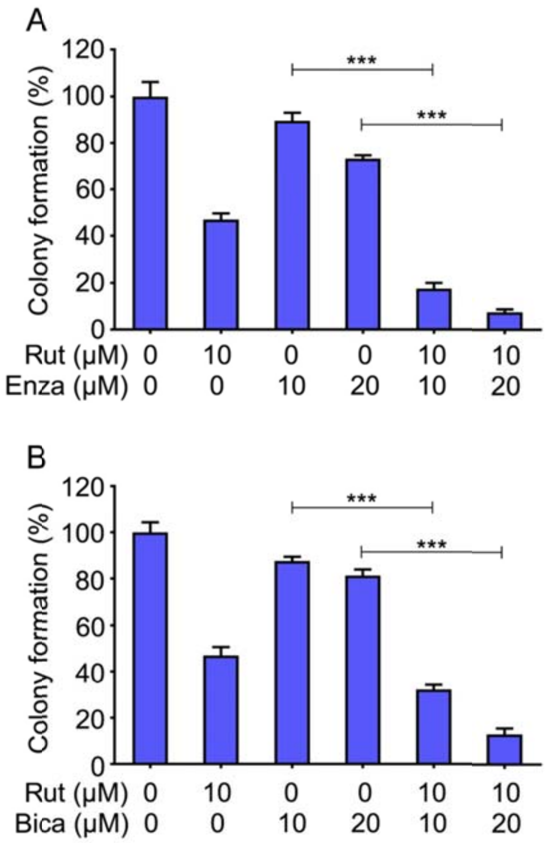

Figure S6. Rutaecarpine suppressed the growth of CRPC in *vitro.* (A-B) Quantitative data of (**Figure 6G-H)** are shown. Mean ± SD (n = 3). ***P<0.001.

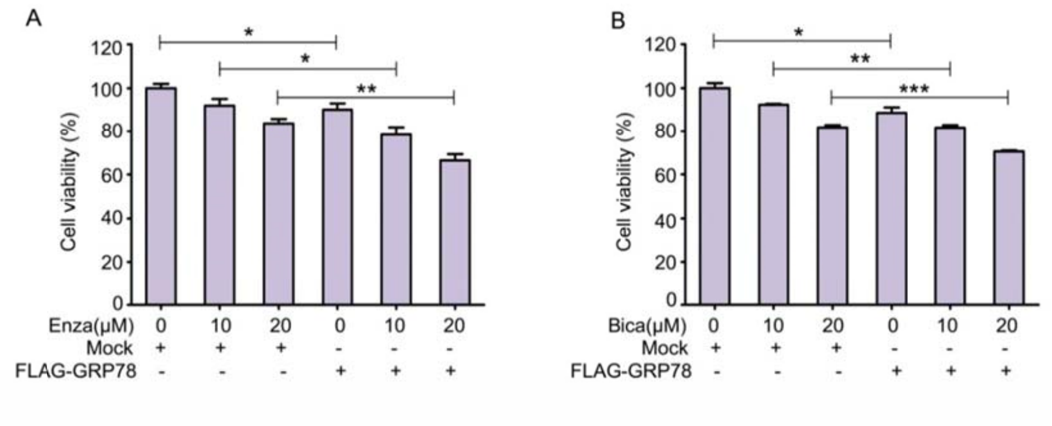

Figure S7. Overexpression of GRP78 enhanced the sensitivity of CRPC cells to the anti-androgen therapeutics in *vitro*. (A-B) MTS assay was performed in 22Rv1 cells treated with FLAG-GRP78 or control plasmid for 24 h, and then exposed to Enzalutamide (Enza) /Bicalutamide (Bica) for 24 h. Mean ± SD (n = 3). *P<0.05, **P<0.01, ***P<0.001.
